# Xeno‐pericardial patch repair of the inferior vena cava for radical resection of renal cell carcinoma with tumor thrombus

**DOI:** 10.1002/jso.24709

**Published:** 2017-06-12

**Authors:** Satsuki Fukushima, Motohide Uemura, Kunihito Gotoh, Takeshi Ujike, Hiroshi Wada, Shigeru Miyagawa, Koichi Toda, Yoshiki Sawa

**Affiliations:** ^1^ Department of Cardiovascular Surgery Osaka University Graduate School of Medicine Suita Osaka Japan; ^2^ Department of Urology Osaka University Graduate School of Medicine Suita Osaka Japan; ^3^ Department of Gastroenterological Surgery Osaka University Graduate School of Medicine Suita Osaka Japan

**Keywords:** IVC tumor thrombus, renal cell carcinoma, surgical team

## Abstract

**Background and Objectives:**

For tumor thrombus in the inferior vena cava (IVC) complicated with kidney cancer, we built a surgical team to achieve (1) en bloc tumor resection; (2) xeno‐pericardial patch IVC repair; and (3) minimum organ damages. We reviewed outcome of the case series to verify rationale of this approach.

**Methods:**

A consecutive series of 12 patients having the IVC tumor thrombus by renal cell carcinoma in the last 3 years was enrolled. Minimum kidney ischemia was induced in five cases (Procedure I), whereas liver and kidney ischemia was induced in five cases (Procedure II). Mild hypothermic extracorporeal circulation was used in two cases (Procedure III).

**Results:**

There was no mortality or severe morbidities related to the surgery. Postoperative recovery was most prompt by the Procedure I. Liver and kidney ischemic time was longer in the Procedure III than the Procedure II, whereas organ function was not substantially impaired in either series. The resected IVC margin was free from the cancer in all cases, while local recurrence was not seen in any cases.

**Conclusions:**

En bloc resection with xeno‐pericardial patch repair of the IVC was successfully performed in the tumor thrombus complicated with kidney cancer with minimum organ damage.

## 1 INTRODUCTION

Surgical resection of renal cell carcinoma complicated by tumor thrombus in the inferior vena cava (IVC) is one of the challenges with reported mortality being 2.7‐10% even in the contemporary series,^1^, ^2^, ^3^ although it reportedly contributes to improvement of symptom and prognosis in more than half of the affected patients.^4^, ^5^, ^6^, ^7^ Keys of surgical success would include (1) complete removal of the malignant tissue by en bloc tumor resection; (2) re‐establishment of the venous return by adequate IVC repair; and (3) minimum surgery‐associated organ damages.^8^ We built a surgical team, comprised of cardiovascular, hepatobiliary, and urogenital surgeons, for these purposes.^1^, ^9^ We herein reviewed the institutional outcome of a contemporary case series of the renal cell carcinoma complicated by IVC tumor thrombus, to verify the rationale of this team approach.

## 2 MATERIALS AND METHODS

### 2.1 Cohort and data collection

The departmental surgical database contained a consecutive series of 12 patients who had an IVC tumor thrombus extending from renal cell carcinoma in Osaka University Hospital between December 2014 and October 2016. Data was collected from medical charts, operation reports, and referral letters in October 2016. All patients gave written informed consent for the surgery and for use of data and sample for diagnostic and research purpose prior to the surgery. Institutional review board gave a waiver to this retrospective study.

### 2.2 Preoperative evaluation and preparation

After the routine staging of the kidney cancer, the patients were examined by multidetector computed tomography cross sectional scanning to assess Novick and Neves classifications^4^ or blood flow in the proximal and the distal IVC.^10^ Surgical indication and procedure were discussed by the surgical team comprised of urogenital, hepatobiliary, and cardiovascular surgeons. Aspirin was promptly given after the diagnosis, replaced by heparin injection at 7 days prior to the surgery. Presurgical oncological therapy was not given apart from the Case 6, who displayed tumor thrombus‐related hepatic dysfunction prior to the surgery.^11^ In this case, sunitinib was effective in normalizing the liver function. As a result, the surgery was performed at average interval of 38 days, raging 11‐471 days, after the diagnosis, while there were no cases that showed substantial progression of the cancer or thromboembolic events during this interval.

### 2.3 Surgical technique and procedure by the multidisciplinary team

In the supine position under general anesthesia, firstly, the hepatobiliary team exposed the intrahepatic IVC with the right side liver mobilization by the “piggy‐back” liver detachment technique, before the right, the middle, and the left hepatic veins were dissected with vessel loops placed.^12^, ^13^ Median sternotomy prior to the liver mobilization helped the exposure in the Case 6 and 7. In addition, the hepatoduodenal ligament was encircled with a vessel tape to permit the Pringle maneuver. Subsequently, the urogenital surgical team dissected the diseased kidney and divided its renal artery. Direct ultrasonography study was performed to make a final decision to select the surgical procedure, before heparin‐sodium (150 U/Kg body weight) was given to achieve activated coagulation time more than 250 s.

**Figure 1:**
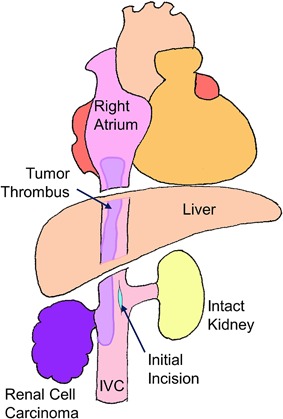
The tip of the tumor thrombus and the area of the IVC wall that was adhered by the tumor thrombus was examined direct ultrasonography study, by which final decision was made to select one of the three surgical procedures. Incision of the IVC was started in the tumor‐free IVC wall at the level of the renal vein, and extended longitudinally to identify the tips of the tumor thrombus with 5 mm tumor‐free margin remaining in the IVC wall

**Figure 2:**
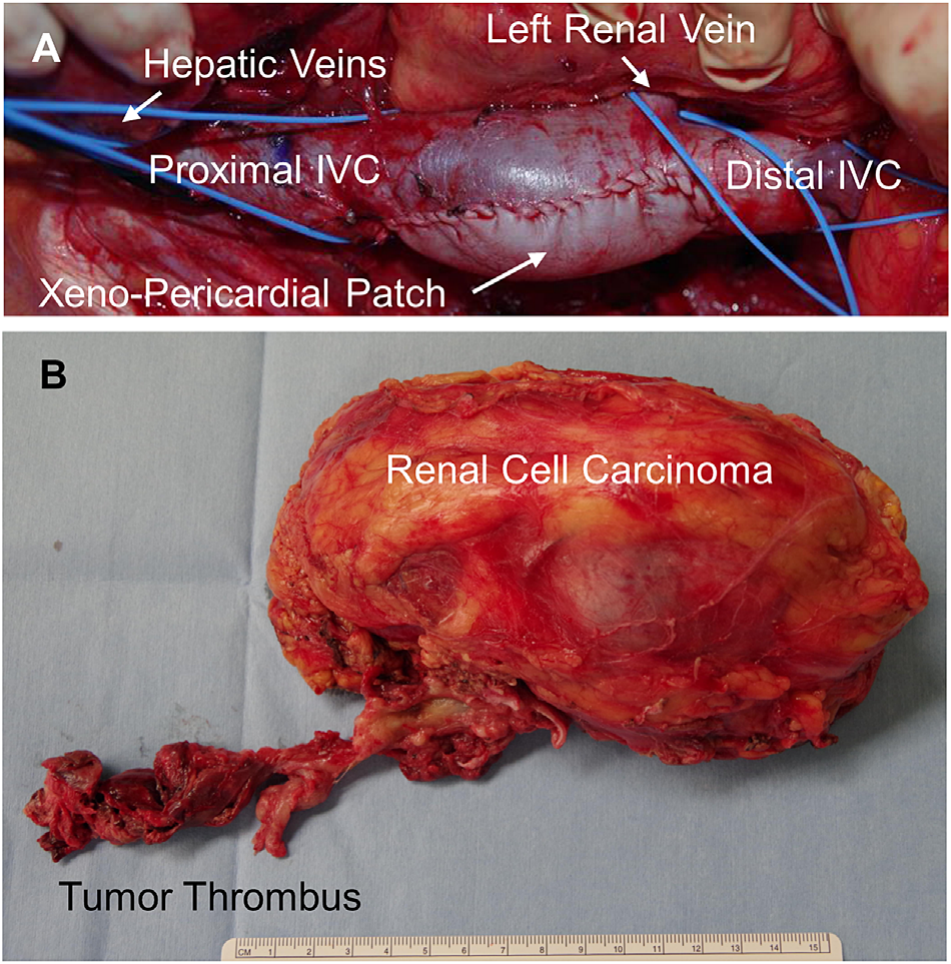
(A) The inferior vena cava (IVC) was repaired by glutaraldehyde‐treated bovine pericardial patch after the removal of IVC tumor thrombus under the view of liver mobilization. (B) The tumor was removed en bloc including the tumor‐thrombus and the diseased kidney

Longitudinal incision of the IVC was started from the tumor adhesion‐free point at the level of the renal vein, which was proximally extended with keeping 5 mm tumor‐free margin. The tumor thrombus showed a dense adhesion to the IVC wall in all cases, so that the affected IVC wall was resected with the 5 mm free margin from the tumor. Once the proximal end of the tumor thrombus was identified, the incision of the IVC was discontinued to remove the tumor with the IVC wall (Fig. 1). Oval‐shaped xeno‐pericardial patch (Edwards Bovine Pericardial Patch, Edwards, Irvine, CA) was used to repair the incised IVC by running suture using polypropylene (5‐0 Prolene, Johnson and Johnson, New Brunswick, NJ, Fig. 2). Since the IVC wall was resected in more than 50% of its circumference in all cases, direct running suture to approximate the incised IVC was avoided not to generate turbulence or disturbance of the venous return. Transesophageal echocardiography was examined intraoperatively in all cases to monitor cardiac function and possible pulmonary artery thrombus by manipulation of the tumor thrombus in the IVC. The procedures were categorized into the following three procedures depending upon the tumor location and the surgical approach for organ protection.

The renal artery of the intact kidney was not clamped in the cases that oblique clamp of the IVC was safe to keep the flow from the renal vein to the distal IVC (Case 4), or that the gonadal vein was large enough to drain the venous flow (Case 5). Otherwise, the renal artery of the intact kidney was clamped to avoid kidney congestion that can cause local hemorrhage or thromboembolism.

### 2.4 Surgical procedure I—infrahepatic IVC reconstruction

**Figure 3:**
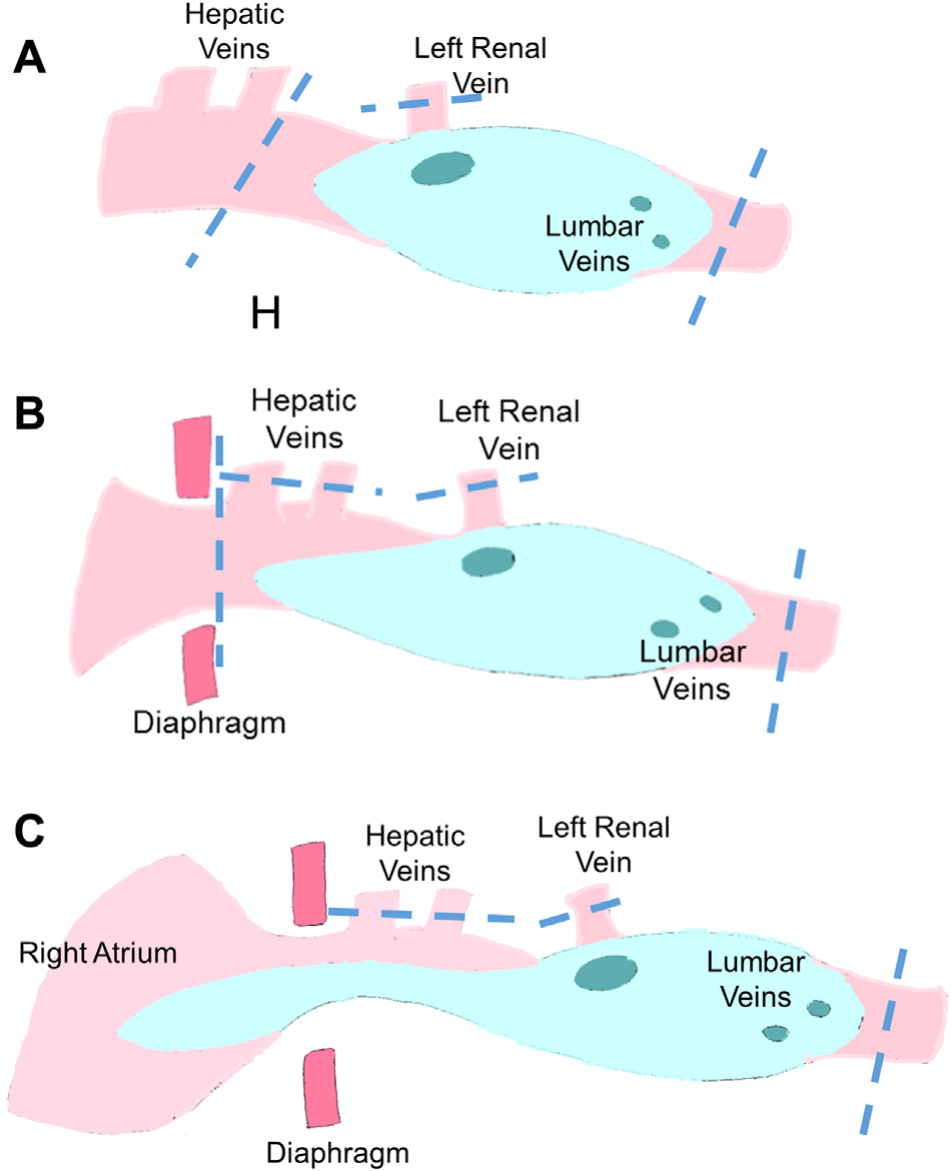
(A) In the cases in which the tip of the tumor thrombus was present below the stem of the hepatic veins, the clamps were placed on the infrahepatic inferior vena cava (IVC), the infrarenal IVC, and the left renal vein, with or without clamp on the left renal artery, to expose the IVC that was longitudinally incised under minimum time of kidney ischemia without liver ischemia (The Procedure I). (B) In the cases in which the tip of the tumor thrombus was present in the intrahepatic IVC, the clamps were placed in the suprahepatic IVC, the hepatic veins, the infrarenal IVC, and the left renal artery/vein to expose the IVC that was longitudinally incised under the liver and the kidney ischemia (The Procedure II). Median sternotomy may be added depending upon the exposure of the suprahepatic IVC. (C) In the cases in which the tumor thrombus was present in the right atrium, extracorporeal circulation with mild hypothermia was established to expose the IVC and the right atrium that was longitudinally incised under induced ischemia in the liver and the left kidney (The Procedure III)

This procedure was indicated for the cases in which the tip of the tumor thrombus was present below the hepatic veins so that infrahepatic IVC was clamped. The clamp of the veins and the artery was placed as following order: (1) the distal IVC; (2) the renal artery; (3) the renal vein; and (4) the proximal IVC (Fig. 3A). After the IVC repair, the clamps were released by the reverse order.

### 2.5 Surgical procedure II—intrahepatic IVC reconstruction

This procedure was indicated for the cases in which the tip of the tumor thrombus was present in the intrahepatic IVC. Depending upon the surgical view, median sternotomy was performed to expose the junction between the IVC and the right atrium. The vessel clamp was placed as following order: (1) the distal IVC; (2) the renal artery; (3) the renal vein; (4) “Pringle”; (5) the hepatic veins; and (6) the suprahepatic IVC to incise the IVC not to damage the hepatic vein orifice (Fig. 3B). When the patch suture reached to the infrahepatic level, where the clamp was re‐placed to release the clamps in the suprahepatic IVC, the hepatic veins, and “Pringle.”

### 2.6 Surgical procedure III—suprahepatic IVC reconstruction

This procedure was indicated for the cases in which the tumor thrombus was present in the right atrium. Median sternotomy with longitudinal incision of the diaphragm was performed to expose the junction between the IVC and the right atrium. After full heparinization, the ascending aorta and the superior vena cava were cannulated to establish extracorporeal circulation with blood temperature kept around 34°C. The vessel clamps were placed as the same order as the Procedure II (Fig. 3C). The IVC was longitudinally incised to extend into the right atrium. After the tumor removal, the atriotomy was directly closed, and the patch suture was started from the stem of the IVC. When the patch suture was completed in the level of the diaphragm, the clamp was placed in the suprahepatic IVC to minimize the blood loss. Subsequently, the patch suture was continued distally as the same way as the Procedure II.

### 2.7 Postoperative treatment and follow‐up

Postoperatively, warfarin was commenced at day 2 post‐surgery and was given for 3 months. Postoperative oncological therapy was commenced, depending upon the pathological stage of the cancer, age or co‐morbidities. The patients visited institutional outpatient clinic of the urogenital surgeon 2‐3 monthly to be reviewed by clinical examinations and blood test and were examined by computed tomography scanning 6 monthly.

## 3 RESULTS

### 3.1 Feasibility and safety of the three surgical procedures

En bloc removal of the tumor was successfully completed in five cases by the Procedure I, in five cases by the Procedure II and in two cases by the Procedure III. The clamps were safely placed on the appropriate position in all cases. The IVC wall was simultaneously resected with the pathologically tumor‐free margin remaining, while the repaired IVC displayed a sufficient blood flow in all cases, examined by intraoperative direct ultrasonography study. (Table 1). All cases recovered postoperatively without serious complications. Follow‐up was completed in all cases October 2016. There were no cases that presented sudden limb edema or pulmonary artery embolism until the latest follow‐up, possibly suggesting absence of complications relating to the repaired IVC postoperatively.

**Table 1:** Preoperative characteristics

	Age	Gender	Clinical stage	Distant metastasis	Novick class	Neves class	Tumor side	Serum creatinine (mg/dL)	eGFR
Procedure I									
Case 1	60	Male	T3bN1M1	Lung	II	I	Right	1.27	46.1
Case 2	68	Male	T3bN0M0	No	II	I	Right	0.99	58.4
Case 3	66	Female	T3aN2M1	Lung	II	II	Right	0.65	69
Case 4	65	Male	T3bN0M0	No	II	II	Right	1.21	47.5
Case 5	69	Male	T4N0M1	Lung	II	II	Right	0.91	63.8
Procedure II									
Case 6	67	Male	T3cN0M0	No	III	III	Right	1.56	35.7
Case 7	80	Female	T3bN0M0	No	II	II	Right	0.52	83.4
Case 8	43	Female	T3bN0M0	No	III	III	Right	0.89	55.3
Case 9	78	Male	T3bN0M0	No	II	II	Right	1.67	31.3
Case 10	65	Female	T3cN0M1	Lung, liver	III	III	Right	0.77	57.6
Procedure III									
Case 11	73	Male	T3cN0M0	No	IV	IV	Right	0.9	63.5
Case 12	72	Male	T3cN0M0	No	IV	IV	Right	0.87	66.2

eGFR; estimated glomerular filtration rate.

### 3.2 Early outcome of the procedures I

The Procedure I was indicated in five cases that displayed the tumor tip present below the hepatic veins, such as Novick classification II (Table 2). Liver mobilization was not performed in the Case 1 and 2 of this group, since extension of the tumor thrombus was limited far below the hepatic veins. The IVC was repaired without liver ischemia in all cases, while short‐time temporary ischemia in the intact kidney was induced in three cases. Resection and reconstruction of the IVC was completed within 30 min in all cases. Postoperative renal function was preserved, achieving a prompt recovery (Table 3).

**Table 2:** Surgical details

					Organ ischaemia (min)				
	Sternotomy	Liver detachment	Additional procedure	IVC clamp (min)	Liver	Kidney	Extra‐ corporeal circulation (min)	Distal IVC	Red blood cell transfusion (Unit)
Procedure I									
Case 1	No	No	No	20	0	5	N/A	Repaired	12
Case 2	No	No	No	15	0	13	N/A	Reparied	0
Case 3	No	Yes	No	22	0	5	N/A	Repaired	4
Case 4	No	Yes	Ascending colectomy	27	0	0	N/A	Reparied	6
Case 5	No	Yes	No	26	0	0	N/A	Repaired	5
Procedure II									
Case 6	Yes	Yes	No	40	24	37	N/A	Stumped	18
Case 7	Yes	Yes	Hepatic artery repair	35	5	30	N/A	Reparied	6
Case 8	No	Yes	No	30	20	30	N/A	Stumped	16
Case 9	No	Yes	No	40	16	29	N/A	Stumped	8
Case 10	No	Yes	Ileocecal resection	35	14	30	N/A	Repaired	16
Procedure III									
Case 11	Yes	Yes	No	50	25	45	79	Repaired	12
Case 12	Yes	Yes	CABG	65	29	60	161	Reparied	28

**Table 3:** Postopetaive outcome

	30‐Day Clavien‐Dindo class	Max ALT (U/L)	Max total bilirubin (mg/dL)	Max creatinine (mg/dL)	Min eGFR	Creatinine at discharge (mg/dL)	eGFR at discharge	Days till discharge
Procedure I								
Case 1	II	92	1.1	1.47	39.3	1.5	38.4	33
Case 2	II	377	0.7	1.41	39.7	0.94	61.8	33
Case 3	II	43	1.7	1.1	38.8	0.9	48.3	18
Case 4	II	31	0.6	2.64	20.2	1.37	41.5	25
Case 5	II	69	0.5	1.25	45.1	1.01	56.9	23
Procedure II								
Case 6	II	213	2	1.77	31.1	1.36	34	30
Case 7	II	523	3.8	1.11	36.4	0.72	58.4	82
Case 8	II	121	1.9	1.14	42.2	0.88	56	72
Case 9	II	76	1	1.85	28.5	1.71	30.9	18
Case 10	II	44	2.8	0.77	57.6	0.88	49.7	25
Procedure III								
Case 11	II	63	1.1	1.24	44.8	0.9	63.5	22
Case 12	II	67	1.7	1.62	33.5	1.02	55.6	47

ALT; alanine transaminase, eGFR; estimated glomerular filtration rate.

### 3.3 Early outcome of the procedures II

The Procedure II was indicated in three cases that were categorized into Novick classification III, in which the tumor tip was located in the intrahepatic IVC (Table 1). The Case 7 and 9 of this group, which were categorized into the Novick classification II, underwent the Procedure II, since the distance between the tumor tip and the hepatic veins was less than 10 mm by the direct ultrasonography. In the Procedure II, the IVC was resected under direct vision of the hepatic vein orifices by temporary liver ischemia.

The liver ischemic time was less than 30 min in all cases, while that of the kidney was less than 40 min (Table 2). The distal IVC was stumped in the Case 6, 8, and 9, since they displayed a complete thrombogenic occlusion of the distal IVC. In these cases, xeno‐pericardial patch repair of the IVC was performed to create adequate venous return from the large lumbar veins and the contralateral intact kidney to the right atrium. As a result, liver and kidney function was temporarily worsened, while they fully recovered (Table 3). In particular, the Case 7, in which the common hepatic artery needed a repair, displayed a substantially worsened liver function postoperatively, but fully recovered. As a result, postoperative recovery was not as prompt in this group as that in the group of the Procedure I.

### 3.4 Early outcome of the procedures III

The Procedure III was indicated in two cases that displayed the tumor extending into the right atrium. The IVC repair was performed under extracorporeal circulation with temporary liver and kidney ischemia. Longitudinal incision of the diaphragm to the IVC provided a clear view of the intrahepatic IVC. The IVC was incised from the renal vein to the right atrium. Since the tumor was densely adhered into the IVC wall in both two cases, resection of the IVC wall was performed to preserve the orifice of the hepatic veins. A long oval‐shape pericardial patch with a 3 cm width was running‐sutured to repair the IVC. The liver ischemic time was less than 30 min in both cases, while that of the kidney was less than 60 min (Table 2). Extracorporeal circulation was ceased when the IVC reconstruction was completed. In the Case 12, coronary artery bypass grafting was performed under the extracorporeal circulation for atherosclerotic stenosis of the right coronary artery prior to the tumor resection. Incised diaphragm was then directly approximated. Postoperatively, either liver or kidney function was not worsened as seen in the group of the Procedure II, although ischemia time in the liver and the kidney was longer than that of the Procedure II group (Table 3). Recovery from the surgery was thus not different from that in the Procedure II group.

### 3.5 Oncological outcome

Pathologically, the resected tumor was renal cell carcinoma in all cases but the Case 4 in which the tumor was diagnosed as Xp11.2 translocation renal cell carcinoma, while resected margin of the IVC wall was free from the malignant tissue in all cases. Stump of the thrombus in the distal IVC in the Case 6, 8, and 9 was a pure thrombus, free from the malignant tissue. Molecular‐targeted therapy by sunitinib was promptly commenced in the Case 1, 3, and 10, who was diagnosed as having distant metastasis preoperatively (Table 4). Sunitinib was also given postoperatively in the Case 8, who showed a tumor recurrence as a distant metastasis at 102 days postoperatively. Recurrence free period was over 500 days in the Case 2, 6, and 11, whereas the Case 8 and 12 were deceased by recurrence within 3 months postoperatively. Local recurrence in the reconstructed IVC was not detected in any cases.

**Table 4:** Oncological outcome

	Postop oncological therapy	Latest status	Cause of death	Follow up post‐surgery (Day)	Recurrence	Tumor free days post‐surgery
Procedure I						
Case 1	Sunitinib	Alive		555	Lung	0
Case 2	No	Alive		527	No	527
Case 3	Sunitinib	Alive		61	Lung	0
Case 4	No	Alive		28	No	28
Case 5	Sunitinib	Alive		14	Lung	0
Procedure II						
Case 6	No	Alive		579	No	579
Case 7	No	Deceased	Pneumonia	223	No	223
Case 8	Sunitinib	Deceased	Recurrence	550	Lung, bone	102
Case 9	No	Alive		377	Bone	277
Case 10	Sunitinib	Alive		403	Lung, liver	0
Procedure III						
Case 11	No	Alive		628	No	628
Case 12	No	Deceased	Recurrence	75	Lung, liver	28

## 4 DISCUSSION

We herein documented a series of renal cell carcinoma with IVC tumor thrombus that were successfully removed en bloc with xeno‐pericardial patch IVC repair by one of the following three surgical approaches. The Procedure I was indicated in five cases in which the proximal tip of the tumor was present far below the hepatic vein level, so that the liver ischemia was not needed, consequently leading to the prompt recovery. The Procedure II was indicated in five cases in which the proximal tip of the tumor was present in the intrahepatic IVC, whereas the Procedure III was indicated in two cases in which that was extended into the right atrium. Temporary liver and kidney ischemia was induced in the Procedure II and III, while extracorporeal circulation was used in the Procedure III. Postoperatively recovery was not different between the Procedure II and III, although ischemic time of the liver and the kidney was longer in the Procedure III than that in the Procedure II. Local recurrence of the tumor was not detected in any cases, while metastatic recurrence was not different in the three groups.

We achieved a complete removal of the malignant tissue by the “en bloc resection,” although one may claim that “sequential step‐resection” would the surgical choice for this pathology. We prefer the “en bloc resection,” since this method would not be surgically demanding to avoid spill and/or remnant of the malignant tissue in the IVC. A clear surgical exposure of the suprahepatic IVC and/or the junction between the IVC and the right atrium was the key of success, particularly in the Procedure II and III.^14^, ^15^ For this purpose, the liver was mobilized routinely and sternotomy was not hesitated regardless of the need of the extracorporeal circulation. In fact, sternotomy was added in the two cases to safely place the clamp in the suprahepatic IVC without extracorporeal circulation.

Novick classification IV, in which the tumor was present in the right atrium, is the surgical challenge even in the contemporary reported series.^3^, ^13^ It was reported that circulatory arrest under deep hypothermia is an option for the Novick IV to expose a blood‐free surgical view of the IVC.^16^ However, in the present series, extracorporeal circulation with heart beating provided an almost blood‐free view of the IVC by adding incision of the diaphragm and liver ischemia. Importantly, the Procedure III did not prolong the recovery as compared to the Procedure II, despite the fact that the organ ischemia time was longer in the cases of the Procedure III than those of the Procedure II. This may be explained by the fact that organ ischemia was induced under mild hypothermia in the Procedure III.

Although there are several reports of the IVC repair by direct suture or by vascular prosthesis,^17^, ^18^ the IVC was repaired by the xeno‐pericardial patch in all cases, First, the repaired IVC needs to be free from blood flow restriction. Second, the xeno‐pericardial patch is composed of the pliable material to be surgically handled and that the it is protective against the local infection which may be caused by gut‐related complications. Moreover, we did not perform the graft interposition in any cases to avoid kinking and/or torsion of the IVC.^15^

Neoadjuvant or adjuvant targeted molecular therapy by using sunitinib has not been established in this pathology with a limited number of reports and inconsistent efficacy.^19^, ^20^ Although we previously used sunitinib prior to the surgery,^11^ the negative results of this presurgical treatment prompted us to move on the postoperative treatments for the patients who had distant metastasis preoperatively or those who had recurrence of the tumor in the distant organ postoperatively.

This study is limited by the retrospective nature and by the small number of the series. Considering the rarity and the advanced stage of this pathology, however, prospective study to compare the surgical approach may not be ethically permitted or effective. In‐depth review of each cases, and/or meta‐analysis would contribute to the care for this pathology.^21^

In conclusion, radical resection and xeno‐pericardial patch IVC repair was successfully performed without surgery‐related mortalities or severe morbidities in the Novick II‐IV tumor thrombus of the IVC complicated with kidney cancer. A clear exposure of the IVC by the surgical team was the key of success to remove the tumor en bloc with a tumor free margin in the IVC with minimum liver and kidney damages.

## SYNOPSIS

This article documents a surgical outcome of 12 contemporary case series of tumor thrombus by renal cancer treated by institutional surgical team that focused on blood‐free surgical view of intrahepatic IVC, which was repaired by oval‐shaped xeno‐pericardial patch. The unique approach contributed successful en bloc resection of the tumor with no surgery‐related mortalities or severe morbidities or no local recurrence until the latest follow‐up.
